# OsASR2 regulates the expression of a defence‐related gene, *Os2H16*, by targeting the GT‐1 *cis*‐element

**DOI:** 10.1111/pbi.12827

**Published:** 2017-10-10

**Authors:** Ning Li, Shutong Wei, Jing Chen, Fangfang Yang, Lingguang Kong, Cuixia Chen, Xinhua Ding, Zhaohui Chu

**Affiliations:** ^1^ State Key Laboratory of Crop Biology College of Agronomy Shandong Agricultural University Taian Shandong China; ^2^ Shandong Provincial Key Laboratory for Biology of Vegetable Disease and Insect Pests College of Plant Protection Shandong Agricultural University Taian Shandong China

**Keywords:** bacterial blight, *cis*‐element, drought, defence response, rice, sheath blight disease

## Abstract

The GT‐1 *cis*‐element widely exists in many plant gene promoters. However, the molecular mechanism that underlies the response of the GT‐1 *cis*‐element to abiotic and biotic stresses remains elusive in rice. We previously isolated a rice short‐chain peptide‐encoding gene, *Os2H16*, and demonstrated that it plays important roles in both disease resistance and drought tolerance. Here, we conducted a promoter assay of *Os2H16* and identified GT‐1 as an important *cis*‐element that mediates *Os2H16* expression in response to pathogen attack and osmotic stress. Using the repeated GT‐1 as bait, we characterized an abscisic acid, stress and ripening 2 (ASR2) protein from yeast‐one hybridization screening. Sequence alignments showed that the carboxy‐terminal domain of OsASR2 containing residues 80–138 was the DNA‐binding domain. Furthermore, we identified that OsASR2 was specifically bound to GT‐1 and activated the expression of the target gene *Os2H16*, as well as *GFP* driven by the chimeric promoter of 2 × GT‐1‐*35S* mini construct. Additionally, the expression of *OsASR2* was elevated by pathogens and osmotic stress challenges. Overexpression of *OsASR2* enhanced the resistance against *Xanthomonas oryzae* pv. *oryzae* and *Rhizoctonia solani*, and tolerance to drought in rice. These results suggest that the interaction between OsASR2 and GT‐1 plays an important role in the crosstalk of the response of rice to biotic and abiotic stresses.

## Introduction

Plants have developed complex mechanisms to defend against pathogen invasion. Upon pathogen recognition, various biochemical events occur in plant cells, such as reactive oxygen species burst, hypersensitive response, antimicrobial peptides and phytoalexins accumulation and callose deposition (Hammond‐Kosack and Jones, 1996; Muthamilarasan and Prasad, 2013). The transcriptional activation of numerous genes upon pathogen infection has been demonstrated. The signal transduction pathways in these activations are of particular interest. It is supposed that these activations are caused by both transcription factors (TFs) and corresponding *cis*‐elements in specific promoter regions (Buscaill and Rivas, 2014; Huang *et al*., 2012). Therefore, an important step of understanding gene activation mechanism is to identify the specific *cis*‐elements that are responsive to external signals.

Currently, studies have shown that many *cis*‐elements are required for the transcriptional regulation of defence genes under biotic stress. The GCC‐box (Muthamilarasan *et al*., 2015; Van der Does *et al*., 2013; Wang *et al*., 2013a) and the W boxes (Gao *et al*., 2016; Liu *et al*., 2016) are two well‐studied pathogen‐inducible *cis*‐elements. The GCC‐box (AGCCGCC) usually locates in the promoters of defence genes (Brown *et al*., 2003; Chakravarthy *et al*., 2003; Zarei *et al*., 2011). Two GCC‐like elements, such as JERE (AGACCGCC) and box S (AGCCACC), have been reported to regulate jasmonate‐ and elicitor‐responsive expression (Kirsch *et al*., 2001; Memelink *et al*., 2001). The W boxes [(T)TGAC(C/T)] are a major class of *cis*‐elements responsible for the induction of many plant genes by pathogen infection (Gao *et al*., 2016; Laloi *et al*., 2004; Lippok *et al*., 2007; Mohr *et al*., 2010; Yamamoto *et al*., 2004). Studies of the *Arabidopsis* transcriptome have also illustrated the importance of the W boxes during systemic acquired resistance (Maleck *et al*., 2000; Petersen *et al*., 2000). In addition to the above two *cis*‐elements, other pathogen‐inducible *cis*‐elements have been found in the defence gene promoters, including PRE2 and PRE4 (Cai *et al*., 2008), G‐box (Alves *et al*., 2013), E‐box (Miyamoto *et al*., 2012), GT‐1 (Park *et al*., 2004) and MYB recognition elements (MREs, Tao *et al*., 2015). However, the regulation mechanisms of these *cis*‐elements in response to pathogen attack remain elusive.

Bacterial blight disease, which is caused by biotrophic pathogen *Xanthomonas oryzae* pv. *oryzae* (*Xoo*), and sheath blight disease, which is caused by the necrotrophic fungus *Rhizoctonia solani*, are the two most common and economically important diseases of rice. To date, over 41 major resistance genes have been identified to against various strains of *Xoo* and some have been characterized (Cheema *et al*., 2008; Guo *et al*., 2010; Miao *et al*., 2010; Verdier *et al*., 2012; Wang *et al*., 2009, 2014; Zhang *et al*., 2014). Moreover, altering the expression of certain transcription factors, such as *OsC3H12* (Deng *et al*., 2012), *OsWRKY45* (Shimono *et al*., 2012) and *TaCPK2‐A* (Geng *et al*., 2013), significantly influenced rice resistance to *Xoo*. Compared with well‐documented studies on resistance against *Xoo*, limited progress has demonstrated that sheath blight resistance was only controlled by minor effect QTLs (Wang *et al*., 2012; Zuo *et al*., 2013). Only a few genes, such as *OsACS2* (Helliwell *et al*., 2013), *Os2H16* (Li *et al*., 2013a), *OsJERF1* (Pan *et al*., 2013) and *OsWRKY4* (Wang *et al*., 2015), were identified as resistant to *R. solani*. However, except an identified *R. solani*‐inducible *cis*‐element (Li *et al*., 2017a), knowledge regarding the regulation mechanisms of *R. solani*‐inducible genes is still very limited.

Previously, we identified a rice defence‐related gene, *Os2H16*, which encoded a short‐chain peptide of unknown function. *Xoo*,* Xanthomonas oryzae* pv. *oryzicola* (*Xoc*) and *R. solani*, as well as abiotic salt or drought stress rapidly induced the expression of this gene. Overexpressing *Os2H16* significantly enhanced rice resistance to bacterial blight disease, sheath blight disease and drought, suggesting that the *Os2H16* promoter is a multipathogen‐inducible and drought‐inducible promoter that contains one or more pathogen‐inducible and drought‐inducible *cis*‐elements (Li *et al*., 2013a). Here, we report that the GT‐1 *cis*‐element (GAAAAA) plays an important role in the *Os2H16* promoter response to pathogen infection and osmotic stress. This finding suggested a novel function for GT‐1 in addition to its salt‐ and pathogen‐inducible activities (Park *et al*., 2004). Furthermore, we demonstrated that the GT‐1 *cis*‐element could be bound to a novel transcription factor, OsASR2. Finally, we showed that altering *OsASR2* expression influences rice resistance to *Xoo*,* R. solani* and drought, which suggests that OsASR2 could modulate the response of rice to pathogen and drought by targeting the GT‐1 *cis*‐element.

## Results

### Putative *cis*‐elements prediction in the *Os2H16* promoter

To characterize the regulatory mechanisms of the *Os2H16* gene, we cloned its promoter region (−2197 to +60). According to the PLACE database (Higo *et al*., 1999), some putative *cis*‐elements are predicted in the *Os2H16* promoter (Figure S1). The TATA‐box (5ˊ‐TATAA‐3ˊ) starts 89‐bp upstream of the ATG and 30‐bp upstream of the transcription start site (TSS). In addition, there was no obvious CAAT box next to the TATA‐box on either strand. The *Os2H16* promoter also contains two GT‐1 elements, GAAAAA (Park *et al*., 2004) on the negative strand, three auxin‐responsive elements, CTTTA and GTCTC (Mironova *et al*., 2014), two GA responsive elements, CCTTTT (Woodger *et al*., 2003) and four ABA responsive elements, ACACG, ACCCG and ACGTG (Li *et al*., 2013b). On the opposite strand of the *Os2H16* promoter, we found one element that is complementary to the TGACG element in association with the as‐1 element (Bacha *et al*., 2015; Krawczyk *et al*., 2002) and six CANNTG elements that are recognized by the helix‐loop‐helix (bHLH) TF superfamily (Wang *et al*., 2013c).

### Tissue‐specific and pathogen‐inducible expression patterns of the *Os2H16* promoter

First, we investigated the tissue expression patterns of the *Os2H16* promoter in transgenic rice. As shown in Figure S2, the *Os2H16* promoter was expressed primarily in young root, anther and endosperm. The GFP fluorescence pattern in transgenic rice plants was similar to the previously reported expression pattern of the *Os2H16* gene (Li *et al*., 2013b).

We then examined the effect of *Xoo*,* Xoc* and *R. solani* on *GFP* reporter gene expression in transgenic rice leaves. Treatment with *Xoo* strain PXO99 activated GFP fluorescence starting at 4 h postinoculation (h p.i.), and this fluorescence was maximized at 12 h p.i. compared with the control. Treatment with *Xoc* strain RS105 resulted in similar GFP fluorescence patterns. *R. solani* strain YWK196 induced *GFP* expression at 12 h p.i., and this expression continued to increase until 24 h p.i. (Figure S3). These results validated that the *Os2H16* promoter could respond to multiple phytopathogens.

### Deletion analysis of the *Os2H16* promoter

**Figure 1:**
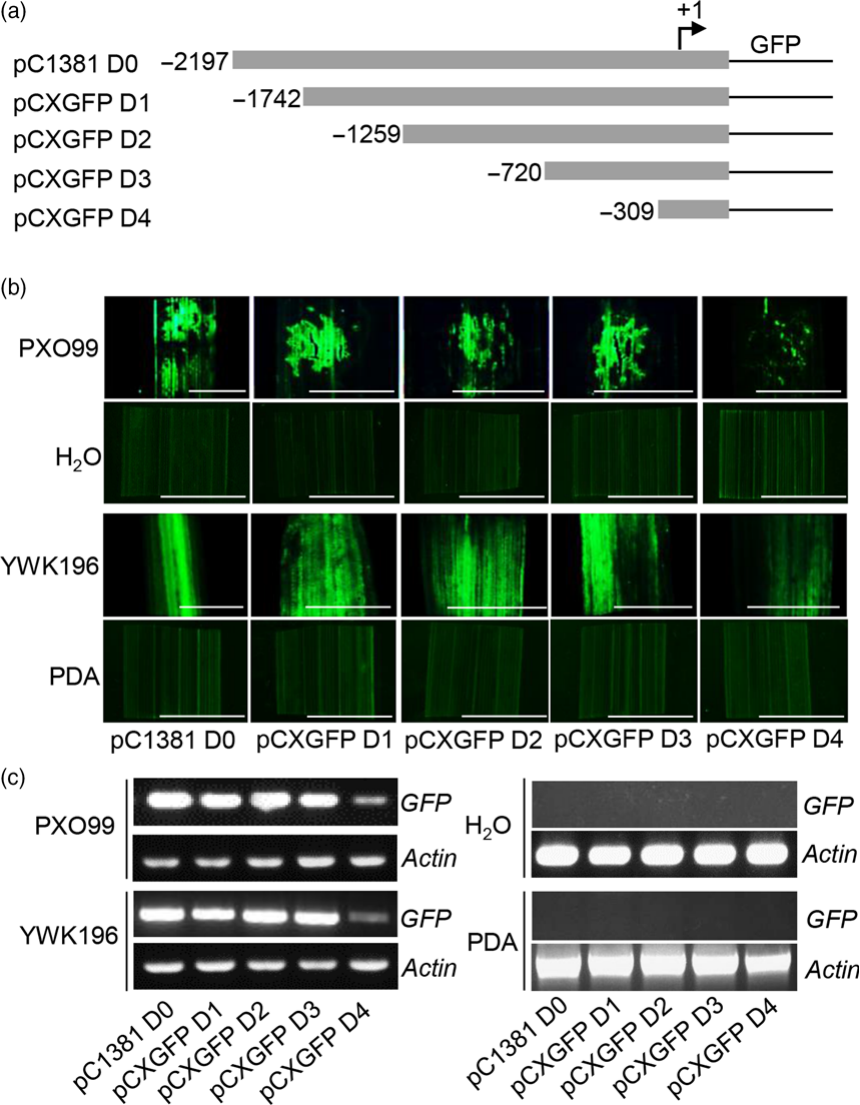
Fluorometric assays for GFP driven by various *Os2H16* promoter deletion constructs. (a) Diagram of various deletion derivatives of the *Os2H16* promoter. Deletion end points are indicated in bp from the transcription start site. All promoter derivatives were fused to a GFP reporter vector, *pCXGFP‐P*. (b) GFP fluorescence of the DNA constructs prepared in (a) in the transgenic rice plants. The rice leaves were inoculated with *Xoo* strain PXO99 and *R. solani* strain YWK196 for 24 h, and no infection was used as control. Bars = 5 mm. (c) *GFP* expression of the DNA constructs prepared in (a) in the transgenic rice plants by RT‐PCR.

To determine the pathogen‐inducible regions in the *Os2H16* promoter, a series of 5ˊ deletions were made in the *Os2H16* promoter (Figure 1a). Each construct was introduced into rice plants by *Agrobacterium tumefaciens*‐mediated transformation, and GFP fluorescence was quantified at 24 h p.i. with *Xoo* strain PXO99 or *R. solani* strain YWK196; no infection was used as a control. The deletion constructs containing up to −1742 (pCXGFP D1), −1259 (pCXGFP D2) and −720 (pCXGFP D3) showed GFP induction almost equal to that of the pC1381 D0 construct. In contrast, GFP inducible activity was nearly lost in the pCXGFP D4 construct containing a deletion up to −309 (Figure 1b,c). These results indicated that the deleted region −720 to −310 contained candidate elements that are essential for the pathogen‐responsive expression of the *Os2H16* promoter.

**Figure 2:**
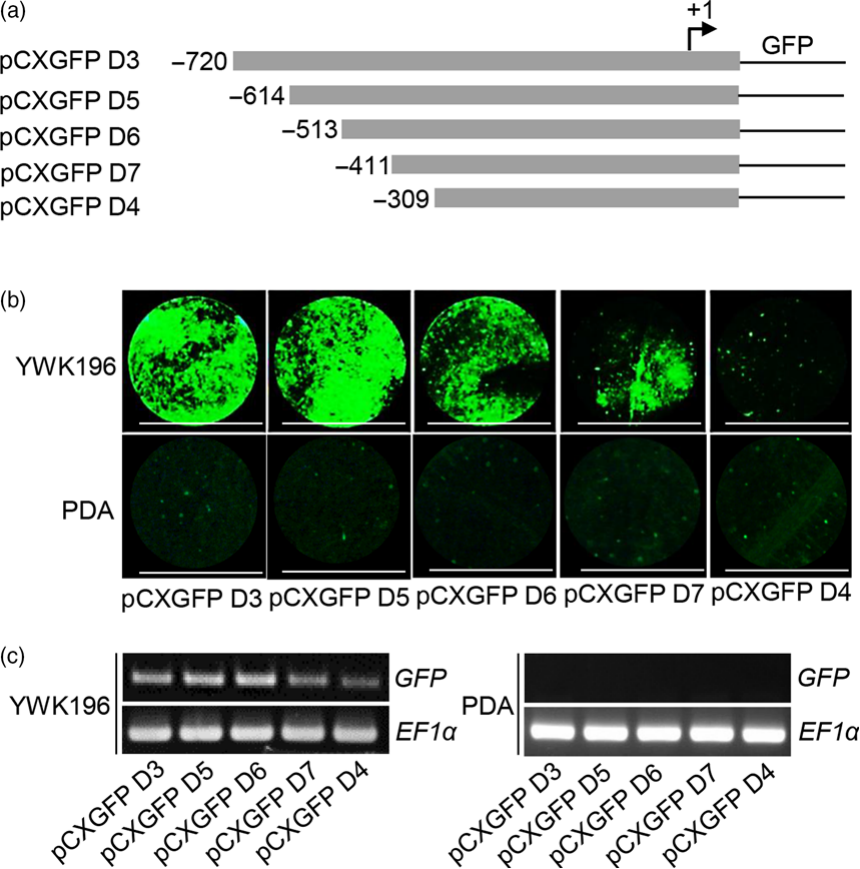
Fluorometric assays for GFP driven by deletion constructs in the −720 to −309 region of the *Os2H16* promoter. (a) Diagram of deletion constructs in the −720 to −309 region of the *Os2H16* promoter. All promoter derivatives were fused to a GFP reporter vector, *pCXGFP‐P*. (b) GFP fluorescence of the DNA constructs prepared in (a) in a tobacco transient expression system. The tobacco leaves were inoculated with *R. solani* strain YWK196 for 24 h, and no infection was used as control. Bars = 5 mm. (c) *GFP* expression of the DNA constructs prepared in (a) in a tobacco transient expression system by RT‐PCR.

To mine the *cis*‐elements within the −720 to −310 region that are responsible for pathogen induction, a series of 5ˊ deletions were generated in this region (Figure 2a). Then, each of construct was tested in transient expression assays in tobacco leaves at 24 h p.i. with YWK196, and no infection was used as a control. The deletion constructs containing up to −614 and −513 (pCXGFP D5 and pCXGFP D6) showed GFP induction almost equal to that of the pCXGFP D3 construct. The construct containing the −411 to +60 region (pCXGFP D7) showed an approximately one‐half reduction in GFP induction compared with the pCXGFP D3, pCXGFP D5 and pCXGFP D6 constructs. However, the deletion of the sequence between −411 and −309 resulted in a significant loss of GFP accumulation (Figure 2b,c). These results demonstrated that two regions, −513 to −412 and −411 to −309, play important roles in the pathogen‐inducible expression of the *Os2H16* promoter, and the −411 to −309 region contained elements that exhibited a more significant effect.

### The GT‐1 *cis*‐element functions importantly in the *Os2H16* promoter response to *Xoo* and *R. solani*

**Figure 3:**
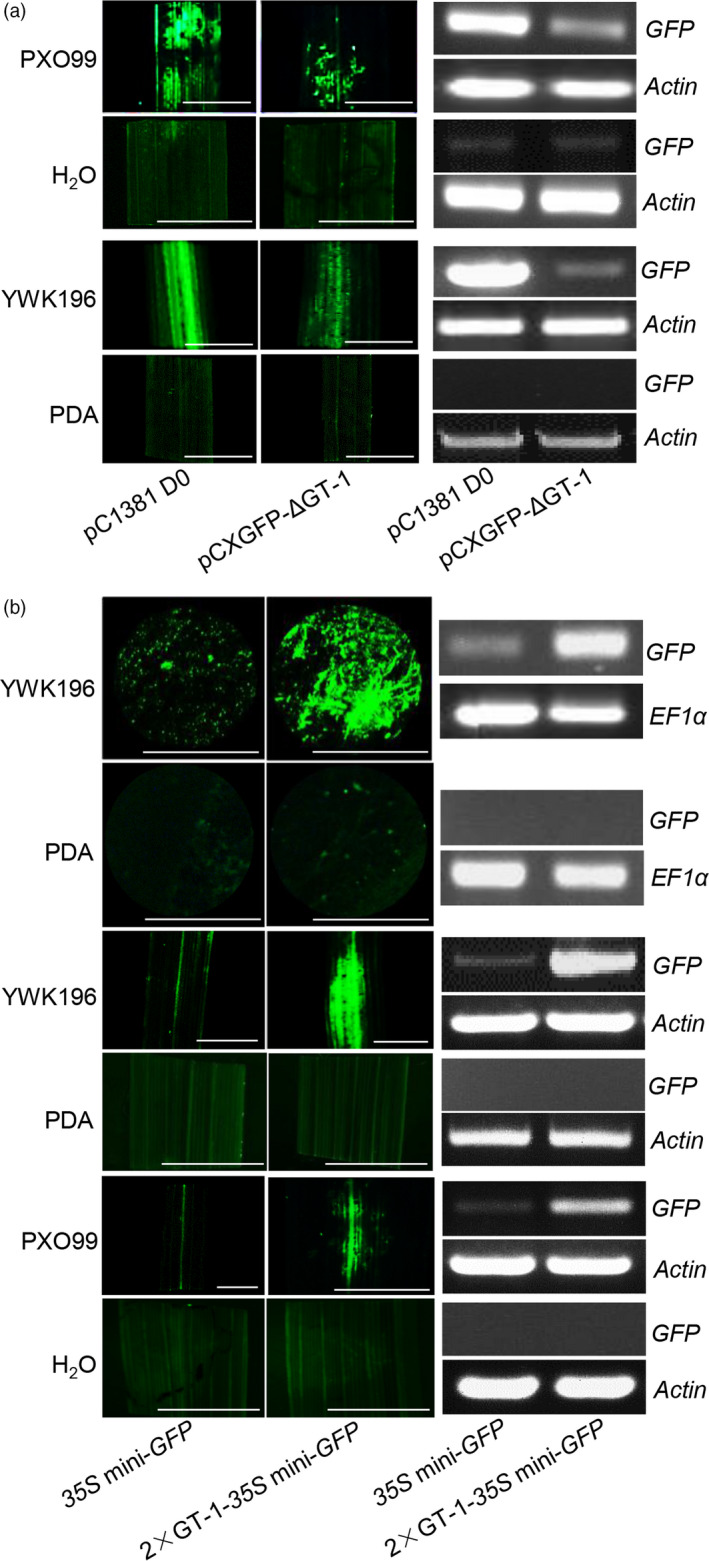
Identification of the GT‐1 *cis*‐element involved in response to pathogens. (a) The effect of deletion of the GT‐1 *cis*‐element within the *Os2H16* promoter on *GFP* expression in the transgenic rice plants. The rice leaves were inoculated with *Xoo* strain PXO99 and *R. solani* strain YWK196 for 24 h and subjected to fluorescence examination and RNA extraction. Bars = 5 mm. (b) The effect of the individual GT‐1 *cis*‐element on *GFP* expression in a tobacco transient expression system and the transgenic rice plants. The tobacco and rice leaves were inoculated with *Xoo* strain PXO99 and *R. solani* strain YWK196 for 24 h and subjected to fluorescence examination and RNA extraction. Bars = 5 mm.

*In vivo* GFP assays of the *Os2H16* promoter in rice and *N. benthamiana* leaves identified two regions, −513 to −412 and −411 to −309, that primarily mediate the pathogen response; the −411 to −309 region more strongly affected the pathogen response than the −513 to −412 region. The −411 to −309 region contained a pathogen‐inducible *cis*‐element, GT‐1 (GAAAAA), which was reported to be responsive to *Pseudomonas syringae* in *Arabidopsis* (Park *et al*., 2004). To test whether GT‐1 plays an important role in the response of the *Os2H16* promoter to *Xoo* or *R. solani*, we made the GT‐1 deletion construct (pCXGFP‐ΔGT‐1) and transformed it into the rice cultivar Zhonghua 11. Transgenic rice plants were then inoculated with PXO99 and YWK196 for 24 h, and no infection was used as control. The *GFP* gene was strongly induced by PXO99 or YWK196 in pC1381 D0 construct, but this induction was significantly reduced in the pCXGFP‐ΔGT‐1 construct (Figure 3a). Therefore, the GT‐1 *cis*‐element plays an important role in the response of the *Os2H16* promoter to *Xoo* and *R. solani*.

To further determine whether the GT‐1 *cis*‐element could respond to *Xoo* and *R. solani* independently, we produced a construct in which two tandem repeats of the GT‐1 *cis*‐element were fused with the *35S* minimum promoter‐*GFP* (2 × GT‐1‐*35S* mini‐*GFP*). The empty vector (*35S* mini‐*GFP*) was used as a control. First, by transient assay in *N. benthamiana* leaves, a large amount of GFP accumulation was observed in leaves expressing the 2 × GT‐1‐*35S* mini‐*GFP* construct, while weak GFP fluorescence was observed in leaves expressing the empty vector (Figure 3b). Furthermore, transgenic rice plants carrying the 2 × GT‐1‐*35S* mini‐*GFP* or *35S* mini‐*GFP* constructs were also generated. These plants were inoculated with PXO99 and YWK196 for 24 h. Compared with the *35S* mini‐*GFP* construct, the *GFP* expression was strongly induced in plants carrying the 2 × GT‐1‐*35S* mini‐*GFP* construct (Figure 3b). These results demonstrated that the GT‐1 *cis*‐element could independently respond to *Xoo* and *R. solani* in rice.

### The GT‐1 *cis*‐element confers drought response

**Figure 4:**
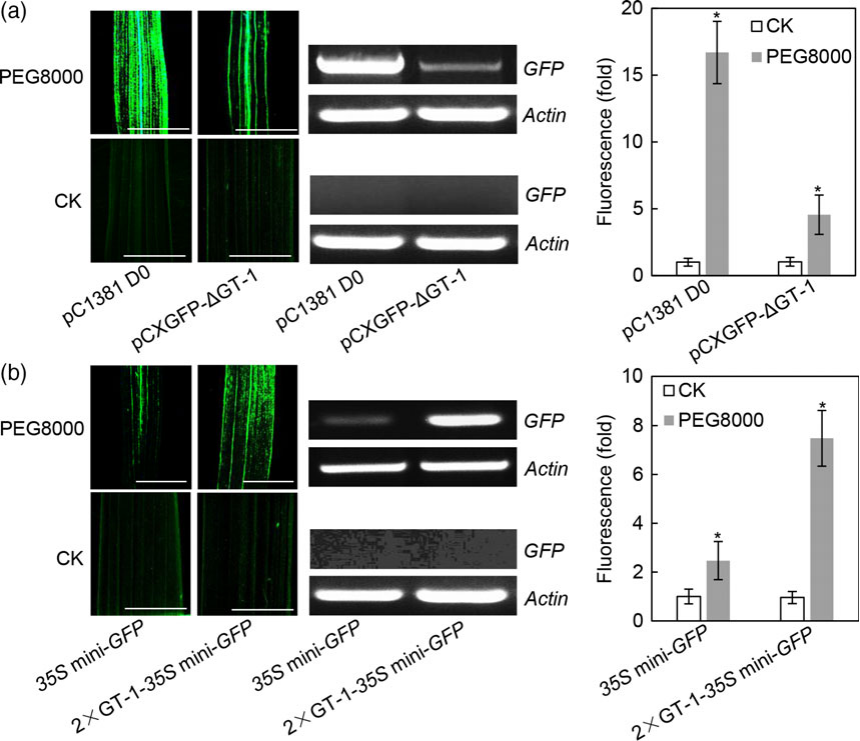
Identification of the GT‐1 cis‐element involved in response to drought. (a) The effect of deletion of the GT‐1 *cis*‐element within *Os2H16* promoter on *GFP* expression in the transgenic rice plants. The rice leaves were treated with PEG8000 (15% w/v) for 24 h and subjected to fluorescence examination and quantification. GFP quantification was calculated relative to CK of the pC1381 D0 construct. Error bars indicate the SD (*n* = 3). Asterisks indicate *P *< 0.05 (*) in Student's *t*‐test analysis. Bars = 5 mm. (b) The effect of the individual GT‐1 *cis*‐element on *GFP* expression in the transgenic rice plants. The rice leaves were treated with PEG‐8000 (15% w/v) for 24 h and subjected to fluorescence examination and quantification. GFP quantification was calculated relative to CK of the *35S* mini‐*GFP* construct. Error bars indicate the SD (*n* = 3). Asterisks indicate *P *< 0.05 (*) in Student's *t*‐test analysis. Bars = 5 mm.

Previous study showed that polyethylene glycol (PEG) could induce the expression of the *Os2H16* gene (Li *et al*., 2013a). To test whether the GT‐1 *cis*‐element could respond to osmotic stress, rice plants transformed with the pCXGFP‐ΔGT‐1 and 2 × GT‐1‐*35S* mini‐*GFP* constructs were subjected to PEG8000 treatment, and no treatment was used as control. As shown in Figure 4a, strong activation of the *GFP* gene was detected in the PEG8000 (15% w/v)‐treated leaves of *Os2H16* promoter transgenic rice, while approximately three quarters of reduction in GFP induction was detected in the pCXGFP‐ΔGT‐1 transgenic rice. This result indicated that the GT‐1 *cis*‐element is partly responsible for the *Os2H16* promoter response to osmotic stress. Moreover, relatively high (threefold) induction of 2 × GT‐1 suggested that the individual GT‐1 *cis*‐element could also respond to osmotic stress (Figure 4b).

### Characterization of a GT‐1 interaction protein

A trihelix DNA‐binding factor, AtGT‐3b, has been reported to bind to the GT‐1 *cis*‐element and regulate gene expression in response to pathogen and salt treatment in *Arabidopsis* (Park *et al*., 2004). To characterize the binding protein of the GT‐1 *cis*‐element in rice, we performed a yeast one‐hybrid (Y1H) analysis to screen the rice cDNA library with GT‐1 as bait. Interestingly, no trihelix DNA‐binding factors were identified from the library, while an abscisic acid, stress and ripening 2 (ASR2) protein was identified and temporarily named OsASR2. OsASR2 contains 138 amino acid residues with a calculated molecular mass of 15.5 kD. Structural analysis showed that it contains an abscisic acid/water deficit stress (ABA/WDS) domain from residues 78 to 128 (Figure S4a). A phylogenetic tree of OsASR2 and other ASR proteins from different plant species showed that it was a highly conserved protein (Figure S4b).

**Figure 5:**
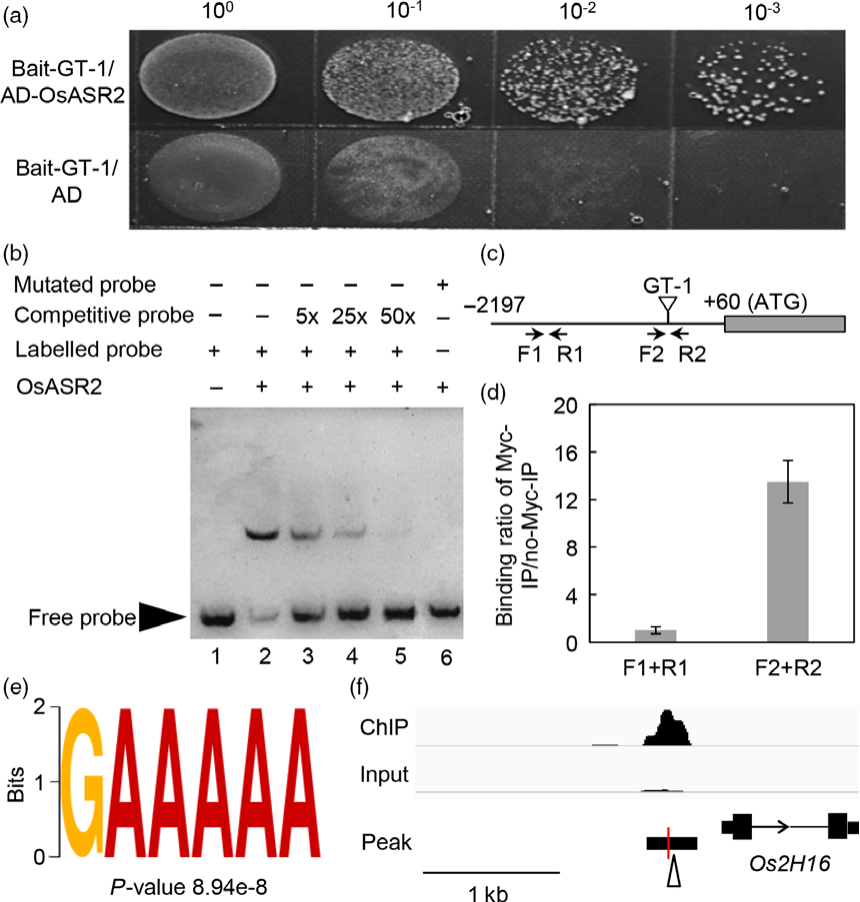
Interaction of OsASR2 with the GT‐1 *cis*‐element. (a) Interaction of OsASR2 with the GT‐1 *cis*‐element by the yeast selection system. Y1HGold yeast strains carrying empty vector *pGADT7* or pGADT7‐OsASR2 were grown overnight at 30 °C. Cultures were diluted to OD_600_ = 1.0, and then, serial 10‐fold dilutions were spotted onto medium lacking Ura and Leu in the presence of 150 ng/mL AbA. (b) EMSA using the recombinant OsASR2 protein and the GT‐1 *cis*‐element. Lane 1 contained only the free probe. Lane 2 contained the purified OsASR2 protein and the probe. For the competitive EMSA, the purified OsASR2 protein was pre‐incubated with 5 (lane 3)‐, 25 (lane 4)‐ or 50 (lane 5)‐fold molar excess of unlabelled GT‐1 *cis*‐element before the addition of the biotin‐labelled GT‐1 *cis*‐element. For the mutant EMSA, the purified OsASR2 protein was incubated with the labelled mutant GT‐1 *cis*‐element. (c) The positions of primers used in the ChIP‐qPCR experiment. (d) Binding of OsASR2 to the GT‐1 *cis*‐element in a ChIP‐qPCR assay. (e) The GT‐1 binding motif identified in OsASR2 binding peaks in ChIP‐seq assay. (f) OsASR2 biding profile in the promoter of *Os2H16*. The open arrowhead refers to the GT‐1 around the peak summit. The red vertical line denotes the peak summit.

This interaction was firstly verified with a yeast selection system. The yeast cells were transformed with *OsASR2* cDNA fused to the GAL4 activation domain and the GT‐1 upstream of the iso‐1‐cytochrome C minimal promoter. The OsASR2 protein and the GT‐1 *cis*‐element conferred Ura^−^ and Leu^−^ selection in the presence of 150 ng/mL aureobasidin A (AbA). In contrast, yeast cells carrying empty vector did not grow on medium lacking Ura and Leu in the presence of 150 ng/mL AbA (Figure 5a).

Then, this interaction was further analysed *in vitro*. We expressed Myc‐tagged OsASR2 protein in *Escherichia coli* and purified the recombinant protein. The ability of the recombinant OsASR2‐Myc fusion protein binding to the GT‐1 *cis*‐element (5′‐GATTAAGATTTTTCCTACCCTA‐3′) was validated by EMSA. As shown in Figure 5b, the protein in lane 2 bound to the oligo DNA molecule with the GT‐1 *cis*‐element. Lanes 3, 4 and 5, which contained fivefold to 50‐fold molar excesses of the unlabelled GT‐1 *cis*‐element, showed that the intensities of the combined bands weakened. To demonstrate whether the GT‐1 *cis*‐element was essential for the sequence‐specific binding activity, we tested a labelled mutant probe in which the TTTTTC sequence was changed to CCCCCA. No binding band was detected (Figure 5b, lane 6).

To confirm the *in vivo* binding of OsASR2 to the GT‐1 *cis*‐element, the recombinant OsASR2‐Myc fusion protein and the pC1381 D0 construct were co‐transformed into *N. benthamiana* leaves. After 5 days, chromatin was harvested and immunoprecipitations were performed using the antibody specific to Myc. In addition, normal mouse IgG was used as nonspecific control. The primers were designed to amplify the sequence of the *Os2H16* promoter with the GT‐1 *cis*‐element. As shown in Figure S5, OsASR2 bound to the promoter region containing the GT‐1 *cis*‐element. Importantly, the immunoprecipitation was specific to OsASR2, because the promoter region was not detected when a nonspecific antibody was used for immunoprecipitation. The above results provide strong evidence for an interaction between OsASR2 and the GT‐1 *cis*‐element.

### OsASR2 is a functional transcription factor

**Figure 6:**
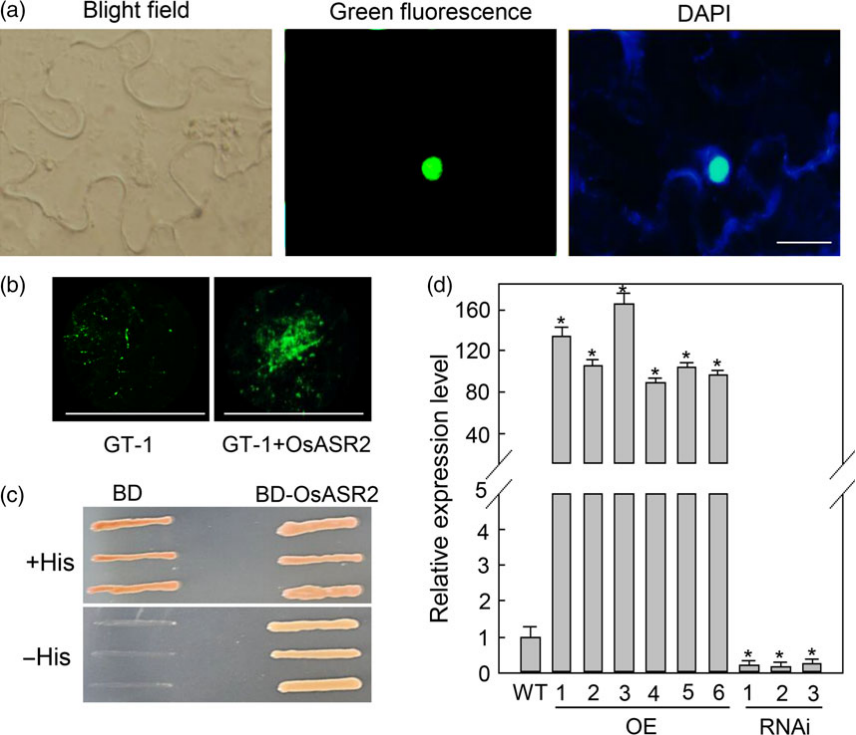
OsASR2 is a transcription factor. (a) Subcellular localization of OsASR2. OsASR2 was fused to GFP to yield OsASR2‐GFP recombinant protein. The construct was transiently expressed in tobacco leaves, and subcellular localization was determined by GFP assay and DAPI staining. Bars = 10 μm. (b) Activation of the GT‐1 *cis*‐element by OsASR2 *in vivo*. GT‐1 alone or GT‐1 and OsASR2 were transformed into tobacco leaves, and GFP was examined by fluorimetric assays. Bars = 5 mm. (c) Transcriptional activation activity of OsASR2. Yeast cultures were diluted and then streaked onto nonselective medium (+His) and selective medium (−His) plates and incubated at 30 °C for 3 days before examination. (d) Expression levels of *Os2H16* in *OsASR2* OE and RNAi lines. Error bars indicate the SD (*n* = 3). Asterisks indicate *P *< 0.05 (*) in Student's *t*‐test analysis.

Transcription factors bind to promoter sequences to regulate the expression of downstream genes, and most of these transcription factors are located in the nucleus. To identify the localization of OsASR2, we constructed an OsASR2‐GFP fusion protein and demonstrated that it exclusively localized to the nuclei of *N. benthamiana* epidermal cells (Figure 6a).

The above results showed that OsASR2 could interact with the GT‐1 *cis*‐element, but the ability of OsASR2 to activate the GT‐1 *cis*‐element remains unknown. To test the specific activation activity of OsASR2, the 2 × GT‐1‐*35S* mini‐*GFP* construct and the pCXUN‐OsASR2‐Myc construct were co‐transformed into *N. benthamiana* leaves. A large amount of GFP accumulation was detected in leaves expressing both the GT‐1 *cis*‐element and OsASR2 compared with the leaves only expressing the GT‐1 *cis*‐element at 5 dpi (Figure 6b). Therefore, OsASR2 could induce the expression of GFP derived under the 2 × GT‐1‐*35S* mini promoter.

To test the transcriptional activation activity of OsASR2, we performed a transcriptional activity assay using a Y1H system. pGBKT7 carrying the completed *OsASR2* cDNA and empty *pGBKT7* serving as a negative control were transformed into the yeast strain AH109. The activity of the *HIS* gene was tested on a medium lacking histidine. In the absence of histidine, the yeast transformed with the construct containing *OsASR2* survived, while yeast containing the empty vector did not (Figure 6c). These results suggested that OsASR2 has transcriptional activation activity.

We constructed six overexpression (OE) and three RNA interference (RNAi) T_0_ lines of *OsASR2* to explore whether OsASR2 could regulate the expression of *Os2H16*, the putative target gene carrying the GT‐1 *cis*‐element in rice. The basal levels of *OsASR2* expression were analysed in 5‐week‐old T_0_ plants using quantitative real‐time PCR (qRT‐PCR) and Western blotting (Figure S6). As shown in Figure 6d, *Os2H16* expression was significantly induced in OE lines and suppressed in RNAi lines compared with WT plants. Therefore, OsASR2 could regulate the expression of *Os2H16* by interacting with the GT‐1 *cis*‐element.

Additionally, the interaction between OsASR2 and the GT‐1 *cis*‐element was validated in transgenic rice through ChIP‐qPCR. Chromatin was harvested, and immunoprecipitations were performed using c‐Myc Tag antibody. In addition, no‐Myc immunoprecipitation was used as a negative control. The primers were designed to amplify the promoter sequences with or without the GT‐1 *cis*‐element (Figure 5c). As shown in Figure 5d, the GT‐1 region was enriched 13.5‐fold compared with the region without the GT‐1 *cis*‐element. To further investigate the OsASR2 binding motifs, the ChIP‐seq assay was performed using the transgenic lines (Figure S7, Accession No. SRP112505). Nine motifs including the GT‐1 *cis*‐element were identified (Figure 5e, Figure S8), and the peak summit for OsASR2 binding site was located 369‐bp upstream of the *Os2H16* TSS (Figure 5f). These results further indicated that OsASR2 could bind to the GT‐1 *cis*‐element.

### Analysis of the *OsASR2* Expression in Rice

To analyse the involvement of OsASR2 in rice basal defence, we examined its expression in response to PXO99 and YWK196. *OsASR2* expression was significantly induced in leaves from 4 h p.i. and peaked at 12 h p.i. but declined to normal levels at 24 h p.i. for PXO99 inoculation. After YWK196 infection, *OsASR2* expression was markedly induced at 12 h p.i. and then declined to a low level but remained higher than that of the control (Figure S9a). Also, treatment with PEG‐8000 (15% v/v) induced *OsASR2* expression at 3 h, which peaked at 6 h and then slightly decreased at 12 h (Figure S9b). This expression pattern indicated that OsASR2 is involved in the pathogen response and osmotic stress.

### Modulating *OsASR2* expression influences rice responses to *Xoo* and *Rhizoctonia solani*

**Figure 7:**
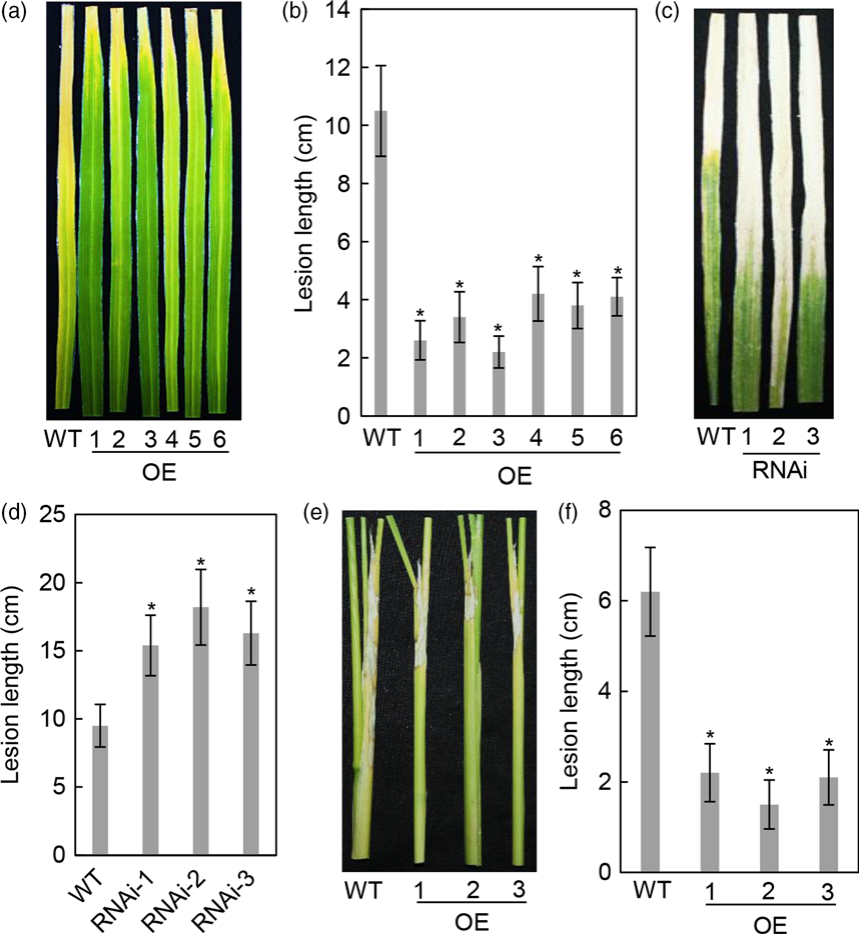
Responses of *OsASR2* transgenic lines to *Xoo* strain PXO99 and *R. solani* strain YWK196. (a) Rice bacterial blight symptoms on WT and T_0_ *OsASR2* OE lines after 21 days of inoculation with *Xoo* strain PXO99. (b) Lesion length after 21 days of inoculation with *Xoo* strain PXO99. Error bars indicate the SD (*n* = 3). Asterisks indicate *P *< 0.05 (*) in Student's *t*‐test analysis. (c) Rice bacterial blight symptoms on WT and T_0_ *OsASR2* RNAi lines after 21 days of inoculation with *Xoo* strain PXO99. (d) Lesion length after 21 days of inoculation with *Xoo* strain PXO99. Error bars indicate the SD (*n* = 3). Asterisks indicate *P *< 0.05 (*) in Student's *t*‐test analysis. (e) Rice sheath blight symptoms on WT and T_1_ *OsASR2* OE lines after 7 days of inoculation with *R. solani* strain YWK196. (f) Lesion length after 7 days of inoculation with *R. solani* strain YWK196. Error bars indicate the SD (*n* = 3). Asterisks indicate *P *< 0.05 (*) in Student's *t*‐test analysis.

To assess the function of OsASR2 in rice disease resistance, we first examined the responses of WT and *OsASR2* transgenic plants to *Xoo* in the T_0_ generation. After 21 dpi with PXO99, six OE lines were enhanced resistance to PXO99, with lesion lengths ranging from 2.2 cm to 4.2 cm, compared with 10.5 cm for WT plants (Figure 7a,b). Three RNAi lines were more susceptible to PXO99, with lesion length ranging from 15.4 cm to 18.2 cm, compared with 9.5 cm for WT plants (Figure 7c,d). The lesion length significantly correlated with the *OsASR2* expression level in OE and RNAi lines, with correlation coefficients of −0.968 (*P *= 0.006) and −0.942 (*P *= 0.009), respectively.

Because *R. solani* could induce *OsASR2* expression, we investigated the role of *OsASR2* in the resistance to *R. solani*. Three T_1_ OE lines were inoculated with YWK196 and investigated further. After 7 days of inoculation, the OE lines showed reductions in lesion length of 4.0–4.7 cm, compared with 6.2 cm for WT plants (Figure 7e,f). These results demonstrated that *OsASR2* confers resistance to both leaf blight bacterium and sheath blight fungus.

### OsASR2 positively regulates rice tolerance to drought stress

**Figure 8:**
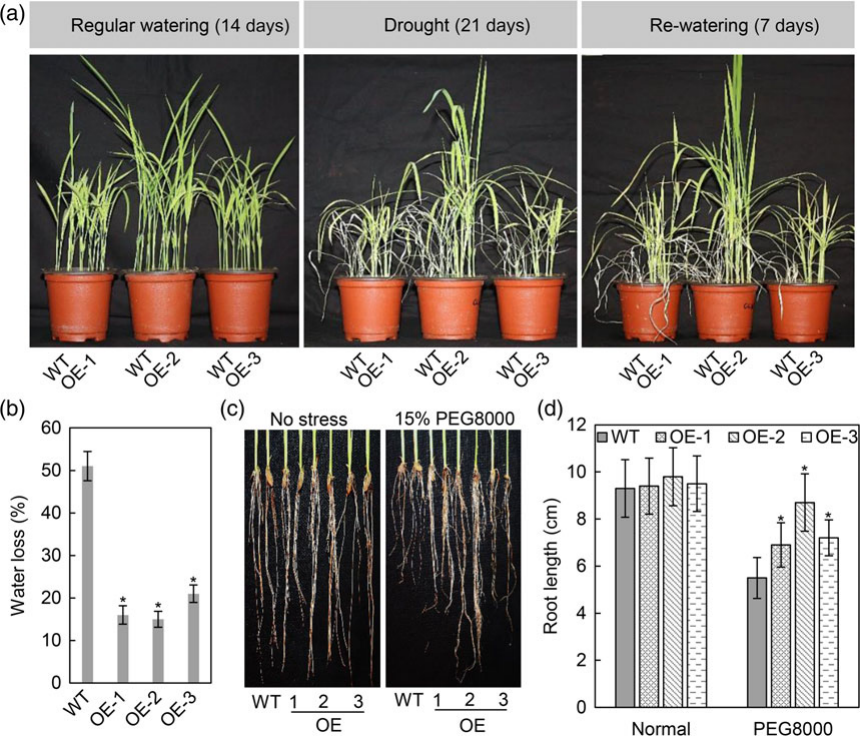
Drought stress tolerance assessment in *OsASR2* transgenic rice plants. (a) Phenotypes of WT and OE lines under regular watering, drought stress and re‐watering conditions. (b) Water loss of three T_1_ OE lines. Error bars indicate the SD (*n* = 3). Asterisks indicate *P *< 0.05 (*) in Student's *t*‐test analysis. (c) Phenotypes of roots from WT and OE lines under normal and osmotic stress. (d) Root length in response to the osmotic stress. Error bars indicate the SD (*n* = 3). Asterisks indicate *P *< 0.05 (*) in Student's *t*‐test analysis.

Due to the induction of *OsASR2* by osmotic stress, we wondered whether *OsASR2* function in drought tolerance. Above three OE lines and WT plants were assayed. When water was cut off, the WT plants exhibited delayed growth compared with the OE lines, which continued growing normally. After drought treatment for 21 days, almost all of the WT plants wilted, while the OE lines exhibited less wilting than WT plants. Upon re‐watering for 7 days, all OE lines recovered but the WT plants did not (Figure 8a). Additionally, the OE lines exhibited a 30%–36% reduction in water loss compared with 51% for WT plants (Figure 8b).

It has been reported that osmotic stress could inhibit root growth (Zhu, 2002). Here, we assessed the root elongation of WT and transgenic seedlings in high osmotic conditions. All OE lines showed little difference in root length compared with WT plants under normal conditions. However, the OE lines displayed longer root lengths than WT plants after treatment with PEG8000 (15% w/v) for 7 days (Figure 8c,d). These results demonstrated that OsASR2 plays a positive role in regulating rice response to drought stress.

## Discussion

Small peptides are the smallest biological molecules mainly containing 5–75 amino acids. They play diverse roles in plant growth, development, reproduction, symbiotic interactions and stress responses (Albert, 2013; Czyzewicz *et al*., 2013; Matsubayashi, 2014). Previously, we identified a small peptide gene *Os2H16*, which was involved in resistance to two important diseases and tolerance to drought (Li *et al*., 2013a). However, the regulatory mechanism of *Os2H16* in the crosstalk of disease and drought resistance remains unclear. In this study, using a promoter deletion analysis, two promoter regions, −513 to −412 and −411 to −309, were proven to be involved in the response of *Os2H16* to pathogen infection (Figure 2). A database search did not identify any known pathogen‐inducible *cis*‐elements in the −513 to −412 region, while a GT‐1 *cis*‐element (GAAAAA) was located in the −411 to −309 region. Moreover, deletion of the GT‐1 *cis*‐element in −411 to −309 region significantly affected the induction levels of the *Os2H16* promoter by pathogens (Figure 3a), suggesting that the GT‐1 *cis*‐element plays a major role in the response of the *Os2H16* promoter to pathogens. Interestingly, deleting the −2197 to −1742 region, which contained the other GT‐1 *cis*‐element (−2137 to −2132), did not affect the pathogen‐inducible activity of the *Os2H16* promoter (Figure 1), implying that the pathogen‐inducible activity of the GT‐1 *cis*‐element might be related to the distance from the TSS.

The *cis*‐element of GT‐1 was first found in the promoter of the ribulose 1, 5‐bisphosphate carboxylase/oxygenase (Rubisco) small subunit gene and identified as the box II elemenτ (Green *et al*., 1987). The GT‐1 *cis*‐elements positively or negatively affect transcription depending on the promoter structure. One common feature of the GT‐1 *cis*‐elements is a core sequence defined as 5ˊ‐G‐Pu‐(T/A)‐A‐A‐(T/A) (Zhou, 1999). The high degeneracy of the GT‐1 *cis*‐element is thought to partly explain its diverse functions except for its light‐specific regulatory function. For example, the GT‐1 *cis*‐element (GGTTAA) is involved in cell‐type‐specific transcriptional regulation (Villain *et al*., 1996), while the GT‐1 *cis*‐element (GAAAAA) responds to *P. syringae* treatment (Park *et al*., 2004). Our results showed that except for *P. syringae*, the GT‐1 *cis*‐element could also be activated by *Xoo* and *R. solani* (Figure 3b), suggesting that it has a broad spectrum of pathogen responses and might be involved in plant basal defence. Additionally, the GT‐1 *cis*‐element reportedly responds to salt stress (Park *et al*., 2004). We found that drought stress could also activate the GT‐1 *cis*‐element (Figure 4b), suggesting a new function for the GT‐1 *cis*‐element in plant response to abiotic stress. Taken together, multiple roles of the GT‐1 *cis*‐element imply its complex mechanisms in plants responding to biotic and abiotic stresses.

In general, binding sites within the promoter and their regulatory proteins control gene expression. The currently identified GT‐1 binding proteins are collectively called GT‐factors, which contain one to three trihelix DNA‐binding motifs (Du *et al*., 2016; Fang *et al*., 2010; Osorio *et al*., 2012). Here, we identified a novel GT‐1 binding protein OsASR2 by yeast one‐hybrid screening, and the interaction between OsASR2 and the GT‐1 *cis*‐element was determined both *in vitro* and *in vivo* (Figure 5, Figure S5). ASR proteins widely exist in plant kingdom, ranging from ancient gymnosperms to monocots and dicots. These proteins are usually nucleic localization and function as transcription factors (Frankel *et al*., 2006; González and Iusem, 2014). Moreover, the DNA‐binding domains of the ASR proteins locate at the carboxy‐terminal end (González and Iusem, 2014) and it has been reported that the amino residues 61–115 of the tomato ASR1 have a DNA‐binding activity (Rom *et al*., 2006). In this study, OsASR2 could activate the expression of the GT‐1 *cis*‐element and the *Os2H16* gene in *N. benthamiana* and rice, respectively, and it was localized in the nucleus and exhibited transcriptional activation activity (Figure 6). Sequence alignments showed that the C terminal (residues 87–138) domain of OsASR2 displayed 65% identities to the DNA‐binding domain of the tomato ASR1 (Figure S10), implying that the amino residues 87–138 might be the DNA‐binding domain of OsASR2. These results strongly suggested that OsASR2 is a transcription factor to regulate the *Os2H16* gene expression by targeting the GT‐1 *cis*‐element.

To date, most studies have focused on the involvement of ASR proteins in sugar transport and ABA response (Dominguez *et al*., 2013; Joo *et al*., 2013; Saumonneau *et al*., 2012), and the roles in improving drought tolerance have also been well characterized (Dai *et al*., 2011; Hu *et al*., 2013, 2014; Li *et al*., 2017b; Liu *et al*., 2010; Philippe *et al*., 2010). However, the involvement of ASR proteins in pathogen response is poorly understood, and the related reports are very rare (Liu *et al*., 2010; Zhu *et al*., 2012). Here, we showed that *OsASR2* could be induced by *Xoo*,* R. solani* and PEG8000 treatments (Figure S9), and overexpressing *OsASR2* significantly enhanced rice resistance to *Xoo*,* R. solani* and drought (Figures 7, 8). Additionally, it has been reported that ASR proteins are crucial during the pollen‐drying stage (González and Iusem, 2014; Wang *et al*., 2013b), and we found that suppressing *OsASR2* expression by RNAi could result in rice abortion in the T_0_ generation. Therefore, we could not test *R. solani* resistance and drought tolerance using RNAi lines in the T_1_ generation. Moreover, a total of 224 genes containing the GT‐1 *cis*‐elements in their promoters were identified and GO analysis showed 83 genes could respond to biotic and abiotic stimulus (Table S2). These results suggest that OsASR2 might positively regulate rice disease resistance and drought tolerance by activating genes containing the GT‐1 *cis*‐element in their promoters.

**Figure 9:**
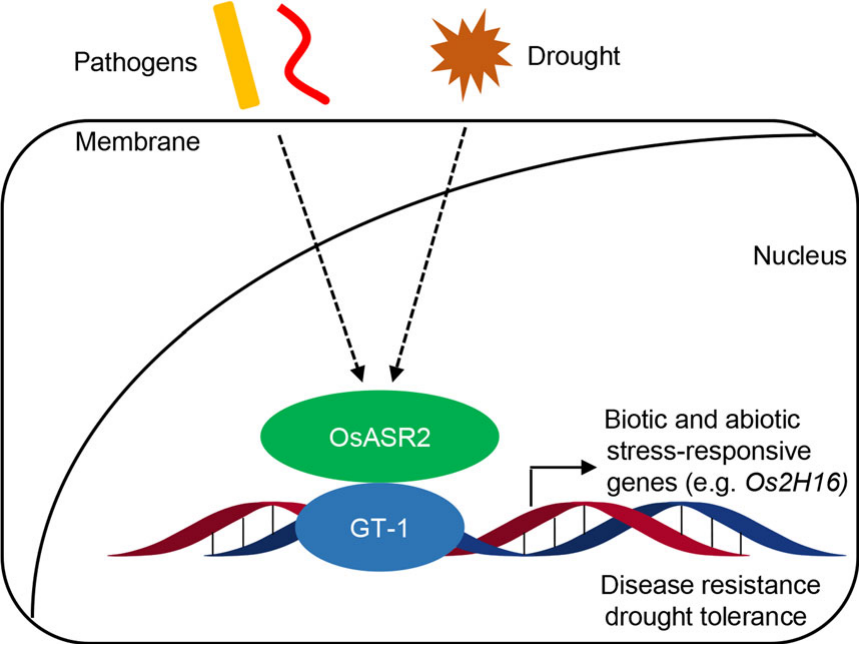
Model for the roles played by DNA–protein interactions in the *Os2H16* gene expression during rice defence responses. First, the OsASR2 transcription factor perceived the both signals of phytopathogens and drought. Then, it activated the expression of the GT‐1 containing genes, such as the *Os2H16*, and finally resulted in disease resistance and drought tolerance.

In summary, we determined the new functions of the GT‐1 *cis*‐element in the response to biotic and abiotic stresses and identified a novel GT‐1 binding protein, OsASR2. Furthermore, we proved that OsASR2 could also respond to pathogens and drought stress and mediate rice resistance to pathogens and drought. The data obtained from this study lead to a model to elucidate the roles played by DNA‐protein interactions in the regulation of pathogen and drought‐responsive genes with GT‐1 *cis*‐element in their promoters, including *Os2H16*, during rice defence responses (Figure 9). First, the OsASR2 transcription factor perceived both signals of phytopathogens and drought; then, it activated the expression of the GT‐1 containing genes, such as *Os2H16*, and finally resulted in disease resistance and drought tolerance.

## Experimental procedures

### Plant materials and treatments

Rice plants (IRBB13 and Zhonghua 11) and *N. benthamiana* were grown at 28 °C and 25 °C with a 16/8‐h light/dark cycle, respectively. For tissue‐specific analysis of the *Os2H16* promoter, tissues from different growth periods of transgenic rice plants were harvested for GFP assay and total RNA extraction. For pathogen‐inducible activity analysis of the *Os2H16* promoter, *Xanthomonas oryzae* strains PXO99 and RS105 were cultured on polypeptone‐sucrose‐agar medium at 28 °C for 2 days and then suspended by sterile water to OD_600_ = 0.5. Plants were inoculated with PXO99 and RS105 suspension by syringe infiltration at seedling stage. YWK196 was cultured on potato‐dextrose‐broth medium at 25 °C for 3 days. Leaves of rice were inoculated with YWK196 and harvested at 4, 8, 12 and 24 h p.i. for GFP assay and RNA extraction (Li *et al*., 2017a). For drought‐induced *OsASR2* expression analysis, roots of 7‐day‐old rice plants were treated with PEG‐8000 (15% w/v). Roots were harvested at 3, 6, 12 and 24 h after treatment for total RNA isolation.

### RT‐PCR and qRT‐PCR analysis

Total RNA was extracted using TRI reagent (Sigma‐Aldrich St. Louis, Missouri, USA) according to the manufacturer's instructions. First‐strand cDNA synthesis was conducted with SuperQuickRT MasterMix Kit (CWBIO, Beijing, China). Quantitative real‐time PCR was performed in an UltraSYBR Mixture (CWBIO) with an ABI QuantStudio™ 6 Flex real‐time PCR detection system. *OsActin* were used to normalize the expression of each gene. Expression changes were calculated using the ΔΔ Ct method. The primers used are listed in Table S1.

### Disease assays

As previously reported (Li *et al*., 2013b), after 21 dpi with *Xoo*, lesion length was measured on T_0_ transgenic lines and WT plants. Lesion length was measured on infected sheaths from transgenic and WT plants at 7 dpi with *R. solani* YWK196. Three independent T_1_ transgenic lines and over ten plants for each line were assessed.

### Drought assays

WT plants and transgenic lines were normally watered for 14 days. Then, water was cut off for 21 days and re‐watering for 7 days (Li *et al*., 2013a). The weights of the OE and WT plants were measured before and after the drought treatment. The water loss was quantified using the following formula, water loss (%) = (fresh weight–dry weight)/fresh weight × 100 (Chen *et al*., 2014). For the root elongation test, the WT and OE seedlings were cultured normally for 14 days. Seedlings were then treated with or without PEG8000 (15% w/v), and the roots length were measured 7 days later.

### Yeast one‐hybrid cDNA library assay, electrophoretic mobility shift assay and chromatin immunoprecipitation sequencing

Yeast one‐hybrid assay was performed with Matchmaker Gold Yeast One‐Hybrid (Y1H) Library Screening System (Clontech, Mountain View, CA) according to the manufacturer's instructions. The probe containing GT‐1 element was synthesized artificially and labelled with dUTP using terminal deoxynucleotidyl transferase (Thermo, Rockford Illinois, USA) and incubated with the purified OsASR2 protein (1 μg per reaction). Subsequently, EMSA was conducted using a LightShift Chemiluminescent EMSA kit according to the manufacturer's instructions (Thermo, Rockford). Chromatin immunoprecipitation (ChIP) was performed with anti‐myc tag antibody‐ChIP grade (Abcam, Cambridge, MA). Library construction and sequencing was performed by Wuhan IGENEBOOK Biotechnology Co., Ltd (http://www.igenebook.com/index.asp). Illumina sequencing libraries were constructed with the OsASR2‐Myc DNA samples according to the manufacturer's instructions. The end of DNA fragments were repaired and ligated to an adaptor. Then, DNA fragment of ~200 bp was selected using SPRIselect beads and amplified by PCR for 18–20 cycles. The amplified DNA products were collected and sequenced with Hiseq2000.

### Statistical analysis

All data analyses were repeated three times with three replicate experiments independently. Standard deviations were indicated by error bars, and the statistical significances were determined by one‐way variance analysis. The mean differences were compared using Student's *t*‐test, and *P* values <0.05 were considered significant.

### Other methods

Details of the methods for plasmid construction and plant transformation, subcellular localization, yeast one‐hybrid, prokaryotic expression, ChIP and transactivation assays are available in supplementary methods (Appendix S1) at *PBJ* online.

## Conflict of interest

The authors declare no conflict of interest.

## Supporting information


**Figure S1** Schematic map of the *Os2H16* promoter with putative promoter elements.
**Figure S2** Tissue expression patterns of the *Os2H16* promoter.
**Figure S3** Pathogen‐inducible expression patterns of the *Os2H16* promoter.
**Figure S4** Analysis of amino acid sequence of OsASR2.
**Figure S5** Binding of OsASR2 to the GT‐1 *cis*‐element in a ChIP assay.
**Figure S6** Verification of *OsASR2* transgenic lines.
**Figure S7** Overview of ChIP‐seq data.
**Figure S8** Binding motifs identified in OsASR2 binding peaks in the ChIP‐seq assay.
**Figure S9** Time causes of *OsASR2* expression in response to pathogen and drought treatments.
**Figure S10** Sequence alignments of OsASR2 with the tomato ASR1.

**Table S1** PCR primers used.

**Table S2** GO analysis of genes containing the GT‐1 *cis*‐element in the ChIP‐seq data.

**Appendix S1** Supplementary methods.
