# Supplementary material for: OsASR2 regulates the expression of a defence‐related gene, Os2H16, by targeting the GT‐1 cis‐element
Source: Plant Biotechnol J. 2017 Oct 10;16(3):771–83. doi: 10.1111/pbi.12827 (PMC5814579; doi:10.1111/pbi.12827)
Supplement: Supplementary file 1 — Figure S1 Schematic map of the Os2H16 promoter with putative promoter elements. Figure S2 Tissue expression patterns of the Os2H16 promoter. Figure S3 Pathogen‐inducible expression patterns of the Os2H16 promoter. Figure S4 Analysis of amino acid sequence of OsASR2. Figure S5 Binding of OsASR2 to the GT‐1 cis‐element in a ChIP assay. Figure S6 Verification of OsASR2 transgenic lines. Figure S7 Overview of ChIP‐seq data. Figure S8 Binding motifs identified in OsASR2 binding peaks in the ChIP‐seq assay. Figure S9 Time causes of OsASR2 expression in response to pathogen and drought treatments. Figure S10 Sequence alignments of OsASR2 with the tomato ASR1. [file PBI-16-771-s001.docx]

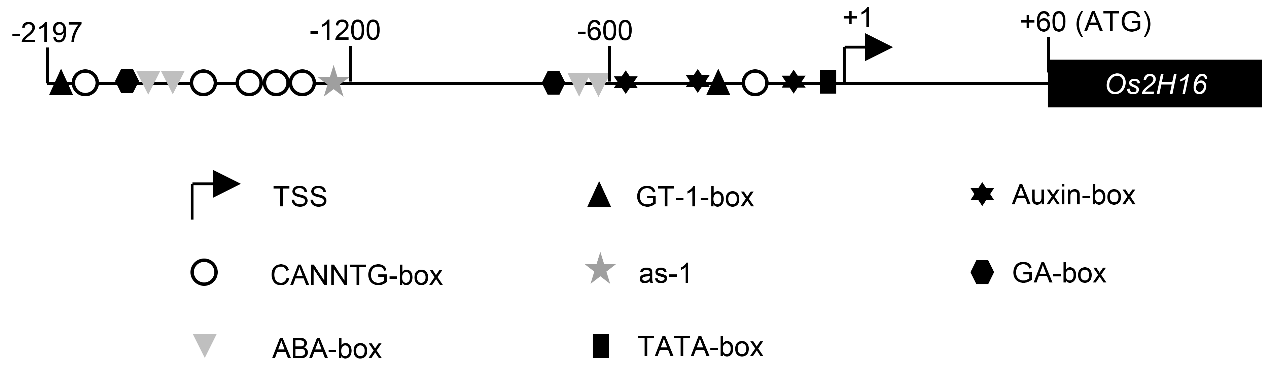


**Figure S1** Schematic map of the *Os2H16* promoter with putative promoter elements. TSS, transcription start site; GT-1-box, pathogen- and NaCl-responsive element; Auxin-box, auxin responsive element; CANNTG-box, nematode responsive box; as-1, box of the CaMV 35S promoter; GA-box, GA responsive elements; ABA-box, ABA responsive elements.


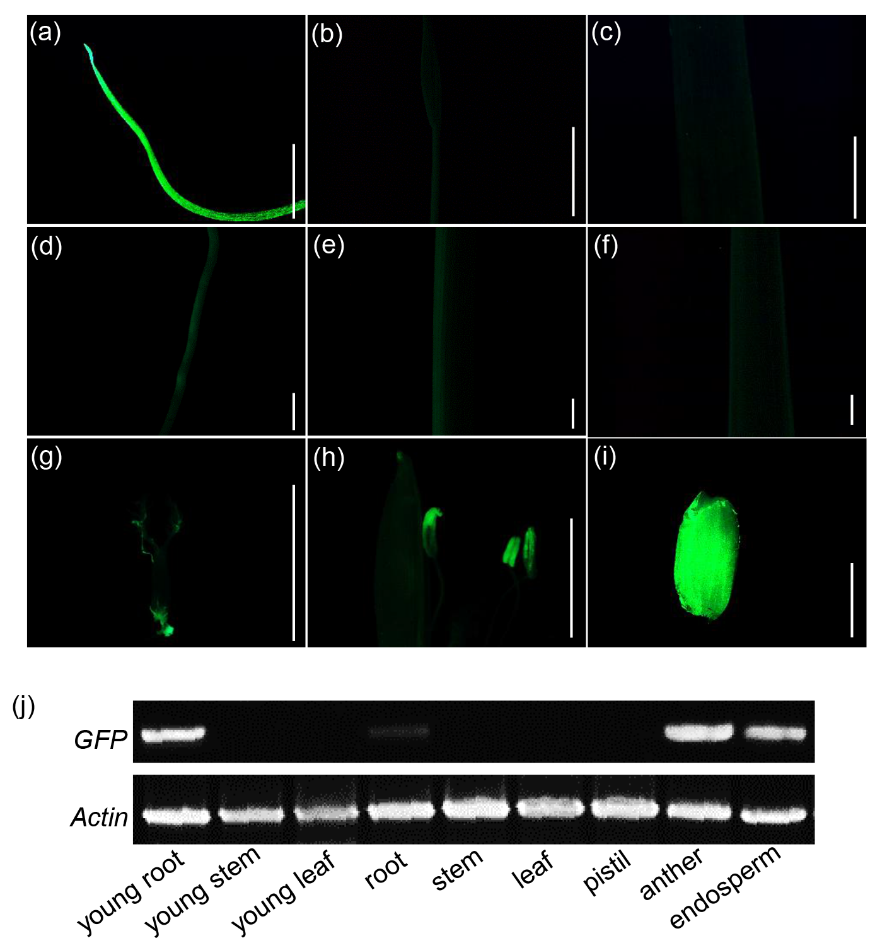


**Figure S2** Tissue expression patterns of the *Os2H16* promoter. (a-i) GFP fluorescence in the *Os2H16* promoter transgenic lines. (a) young root; (b) young stem; (c) young leaf; (d) root; (e) stem; (f) leaf; (g) pistil; (h) anther; (i) endosperm. Bars = 5 mm. (j) Quantitative GFP assays of different tissues from transgenic rice by RT-PCR.


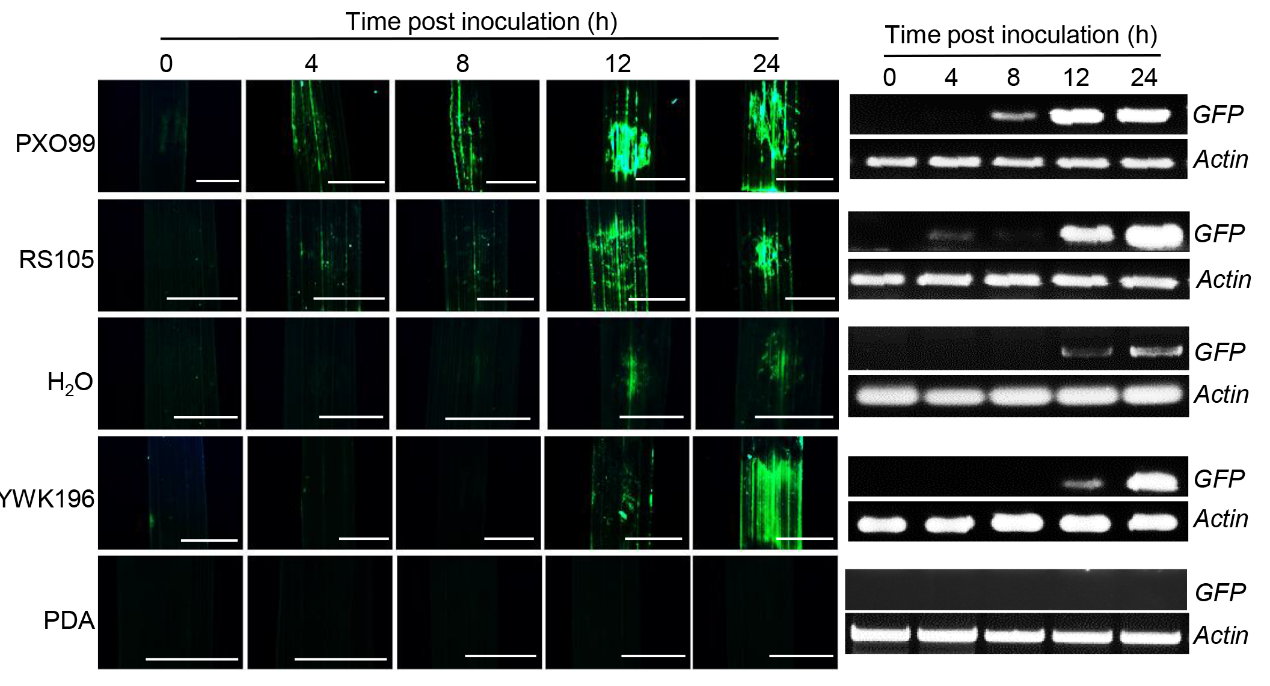


**Figure S3** Pathogen-inducible expression patterns of the *Os2H16* promoter. 21-day-old seedlings were inoculated with *Xoo* strain PXO99, *Xoc* strain RS105 and *R. solani* strain YWK196, H_2_O and PDA broth were used as controls, respectively. The rice leaves were harvested at the indicated time and subjected to fluorescence examination and RT-PCR. Bars = 5 mm.


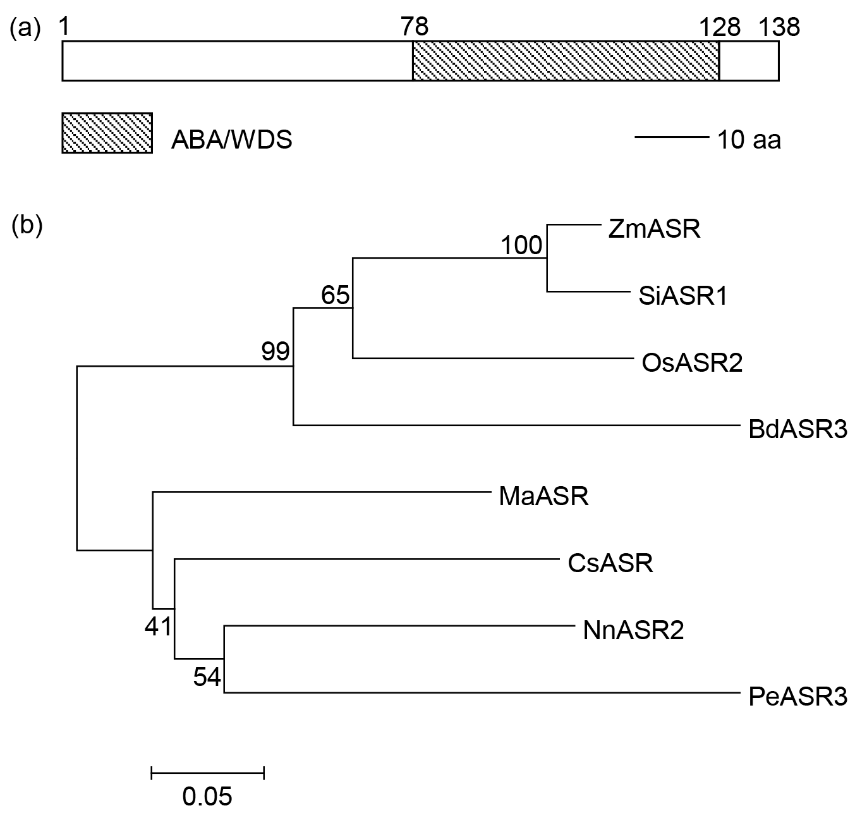


**Figure S4** Analysis of amino acid sequence of OsASR2. (a) Schematic structure of OsASR2. (b) A phylogenetic tree was constructed using MEGA 7 with the neighbor-joining method. The bootstrap values reported for each branch reflect the percentage of 1000 trees containing that branch.


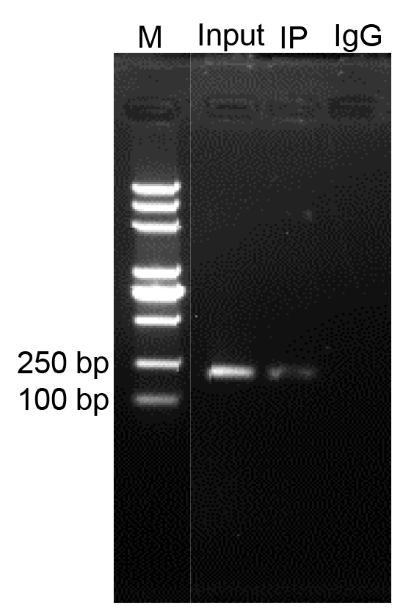


**Figure S5** Binding of OsASR2 to the GT-1 *cis*-element in a ChIP assay. The tobacco leaves were co-transformed with OsASR2-Myc protein and the *Os2H16* promoter. ChIP was performed with an antibody specific for Myc as well as a nonspecific anti-mouse IgG.


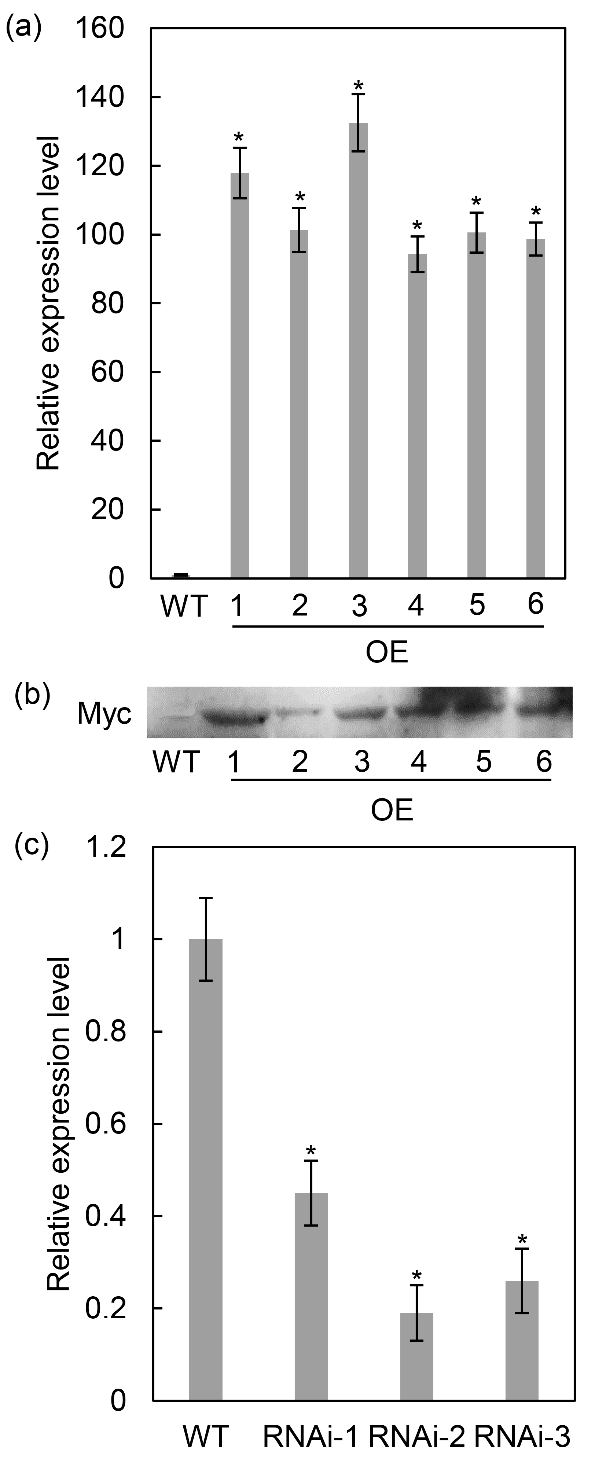


**Figure S6** Verification of *OsASR2* transgenic lines. (a) Expression levels of *OsASR2* in T_0_ OE lines. Error bars indicate the SD (n=3). Asterisks indicate *P* < 0.05 (*) in Student’s t test analysis. (b) Protein levels of OsASR2 in WT and T_0_ OE lines by Western blotting. Proteins of WT and six OE lines was extracted and Western blotting was performed using the antibody specific to Myc. (c) Expression levels of *OsASR2* in T_0_ RNAi lines. Error bars indicate the SD (n=3). Asterisks indicate *P* < 0.05 (*) in Student’s t test analysis.


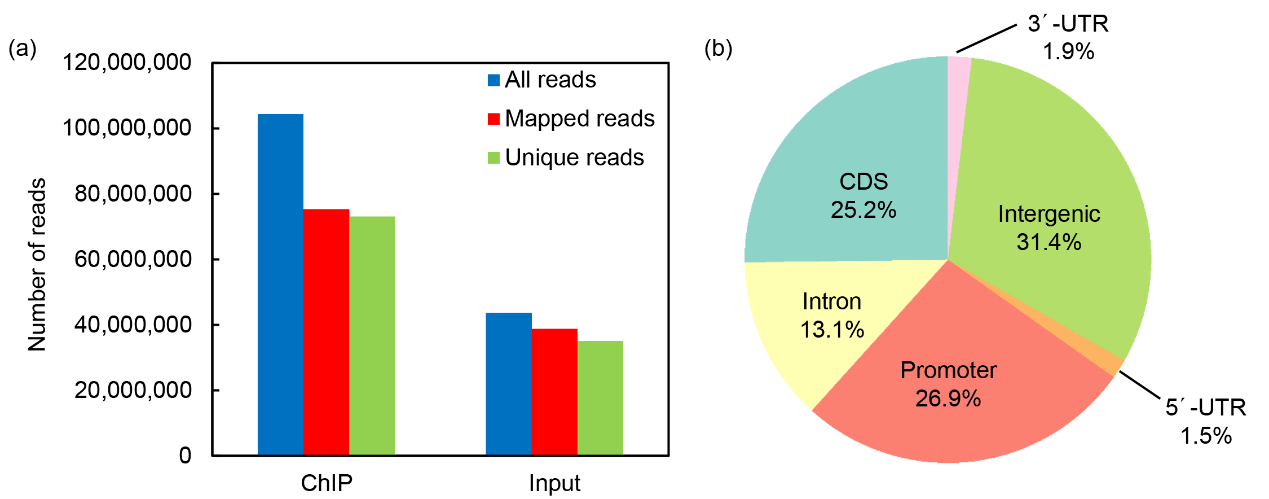


**Figure S7** Overview of ChIP-seq data. (a) Number of all reads, mapped reads and unique reads in ChIP-seq data. (b) Distribution of OsASR2 binding peaks in rice genome.


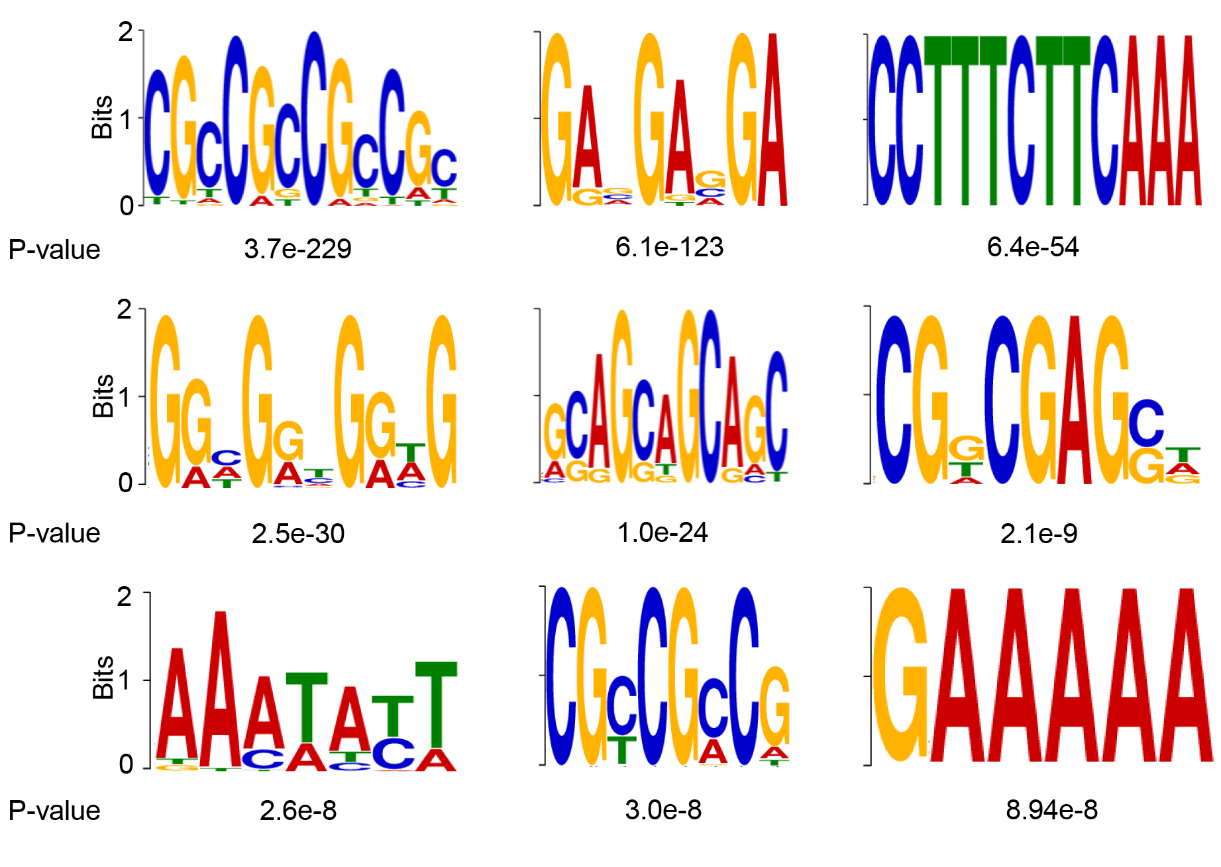


**Figure S8** Binding motifs identified in OsASR2 binding peaks in the ChIP-seq assay.


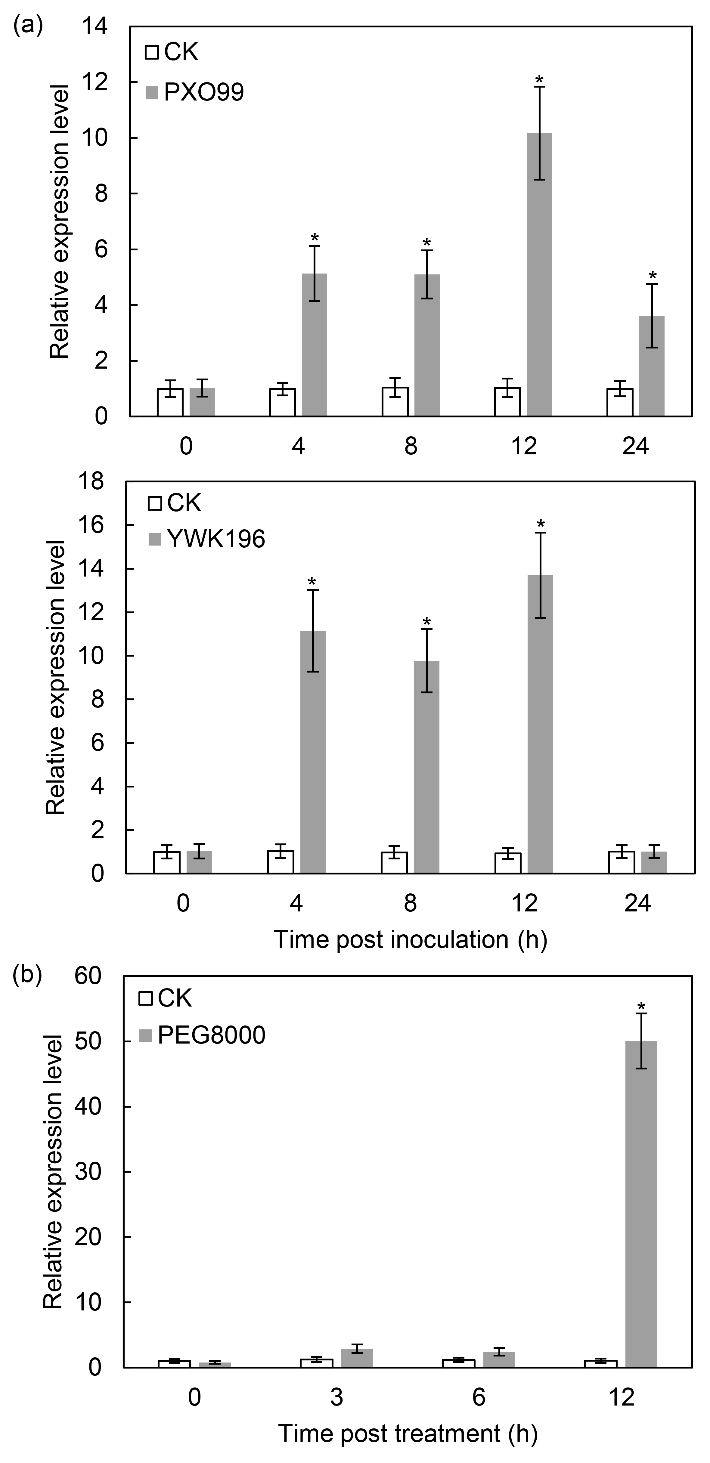


**Figure S9** Time causes of *OsASR2* expression in response to pathogen and drought treatments. (a) *OsASR2* expression under *Xoo* and *R. solani* infection. Rice leaves were inoculated with *Xoo* strain PXO99 and *R. solani* strain YWK196, respectively, and harvested at indicated times for RNA extraction. (b) Expression level of *OsASR2* induced by PEG-8000 treatment. 14-day-old rice seedlings were treated with PEG8000 (15 % w/v), and leaves were harvested at indicated times for RNA extraction. Error bars indicate the SD (n=3). Asterisks indicate *P* < 0.05 (*) in Student’s t test analysis.


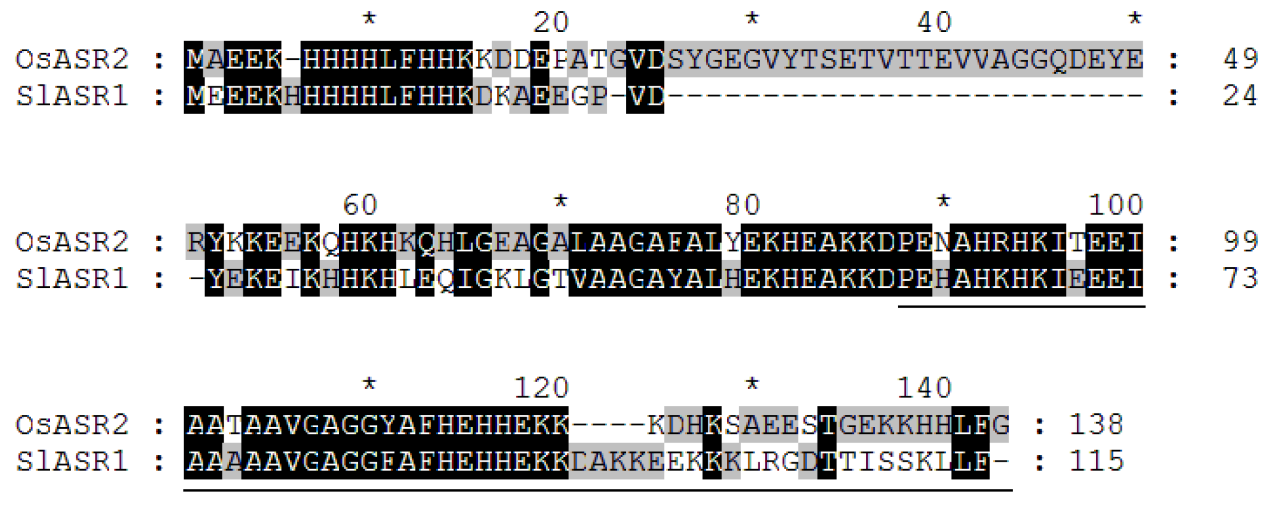


**Figure S10** Sequence alignments of OsASR2 with the tomato ASR1. The DNA-binding domain is underlined.
