# Supplementary material for: OsASR2 regulates the expression of a defence‐related gene, Os2H16, by targeting the GT‐1 cis‐element
Source: Plant Biotechnol J. 2017 Oct 10;16(3):771–83. doi: 10.1111/pbi.12827 (PMC5814579; doi:10.1111/pbi.12827)
Supplement: Supplementary file 4 — Appendix S1 Supplementary methods. [file PBI-16-771-s002.docx]

**Supplementary methods**

**Vector Construction of the *Os2H16* Promoter and *OsASR2***

The full length *Os2H16* promoter containing the -2197 to +60 fragment was amplified from the rice cultivar IRBB13 using primers listed in Table S1. To generate the promoter expression construct, the appropriate restriction sites were introduced into the PCR-amplified promoter (*Sal*I at the 5ˊ end; *Pst*I at the 3ˊ end). The PCR-amplified promoter was then cloned into the *Sal*I/*Pst*I-digested *pCAMBIA1381* to obtain the expression construct, which was named pC1381 D0. The deleted promoters were cloned into the *pCXGFP-P* vector (Chen *et al.*, 2009) to generate deletion constructs containing various fragments (-1724 to +60, pCXGFP D1; -1259 to +60, pCXGFP D2; -720 to +60, pCXGFP D3; -309 to +60, pCXGFP D4; -641 to +60, pCXGFP D5; -513 to +60, pCXGFP D6; -411 to +60, pCXGFP D7). To characterize the GT-1 *cis*-element, the *Os2H16* promoter lacking GT-1 was cloned into the *pCXGFP-P* vector, which was named pCXGFP-ΔGT-1, and GT-1 was repeated twice, ligated into the region upstream of the *35S* minimum promoter within the *pCXGFP-P* vector and named 2 × GT-1-*35S* mini-*GFP*.

To generate the constitutive expression construct of *OsASR2*, the PCR-amplified *OsASR2* cDNA was T-A cloned into the *pCXUN-Myc* vector to obtain the pCXUN-OsASR2-Myc construct. For the knockdown construct, appropriate restriction sites were introduced into the PCR-amplified cDNA suitable for cloning steps (*Spe*I and *Kpn*I at the 5´end; *Sac*I and *Bam*HI at the 3´end). The PCR-amplified part of *OsASR2* cDNA was firstly cloned into the *Kpn*I/*Bam*HI-digested ds1301 and then into the *Spe*I/*Sac*I-digested ds1301 to obtain the ds1301-OsASR2 construct. All primers used for cloning are listed in Table S1. All PCR reactions were conducted with Cobuddy Super Fidelity DNA Polymerase (CWBIO, China).

**Transient Expression in Tobacco and Quantification of GFP**

Promoter analysis was performed by transient expression in tobacco leaves according to a previously described method (Walter *et al.*, 2004). GFP fluorescence was observed under a Leica M205 C stereo microscope and fluorescence was quantified using a Synergy 2 Multi-Mode Reader as described in a previous method (McLellan *et al.*, 2013). GFP fluorescence was excited at 480 nm and measured at 520 nm. pCXGFP D3, pCXGFP D4, pCXGFP D5, pCXGFP D6, pCXGFP D7 and 2 × GT-1-*35S* mini-*GFP* constructs were transiently expressed in tobacco leaves in this study.

**Rice Transformation**

For rice transformation, embryonic callus derived from mature embryos was infected by *A. tumefaciens* EHA105 strain (Lin and Zhang, 2005). For promoter analysis, pC1381 D0, pCXGFP D1, pCXGFP D2, pCXGFP D3, pCXGFP D4, pCXGFP-ΔGT-1 and 2 × GT-1-*35S* mini-*GFP* constructs were transformed into rice cultivar Zhonghua 11 by *A. tumefaciens*-mediated transformation. For gene functional analysis, the pCXUN-OsASR2-Myc and ds1301-OsASR2 constructs were transformed into rice cultivar Zhonghua 11 to generate the transgenic plants.

**Yeast One-Hybrid Assay**

Yeast one-hybrid techniques were performed according to the manufacturer’s instructions (Clontech, Mountain View, CA, USA). The GT-1 *cis*-element sequence was artificially synthesized, and the full-length cDNA fragment of *OsASR2* was amplified. They were cloned into pAbAi and pGADT7 vectors via the restriction sites *Sal*I/*Kpn*I or *Bam*HI/*Sac*I, respectively. Yeast cells were co-transformed with the two plasmid combinations, followed by the selection of transformants on SD medium lacking Ura and Leu for three days at 30°C, and subsequent transfer to medium lacking Ura and Leu in the presence of 150 ng/mL AbA for growth analysis.

**Prokaryotic Expression Analysis**

The rice OsASR2 coding sequence was amplified from IRBB13 cDNA and subcloned into the expression vector *pET-32a* (Novagen, WI, USA) between the *Bam*HI and *Eco*RI sites. The resulting plasmid was transformed into Rosetta^TM^ (DE3) *E. coli* strain BL21 and the transformed cells were grown at 37°C in LB medium overnight at 200 rpm in a shaker. 10 mL aliquot from the overnight culture was added into 1 L of fresh LB medium for 1 h till the OD_600_ reached 0.6-0.8. These cells were induced using 1 mM isopropyl-1-thio-*β*-galactopyranoside (IPTG). After 6 h incubation, the cells were harvested by centrifugation at 5000 rpm for 5 min at 4°C. The pellet was washed with fresh 1 × phosphate-buffered saline (PBS) buffer. Subsequently, the first lysis buffer (300 mM NaCl, 50 mM NaHPO_4_, 10 mM imidazole, 15 % glycerol, and 0.1 % SDS) was added and mixed homogeneously. The cells were lysed by sonication at 6 × pulse for 5 min at 4°C (every 20 s pulse, 10 s interval was given). The lysate was centrifuged at 12000 rpm for 20 min to remove the debris. The supernatant was mixed with the second lysis buffer (300 mM NaCl, 50 mM NaHPO_4_, and 10 mM imidazole) along with 2 mL Ni-NTA, and the volume was made up to 50 mL/L culture of cells and mixed for 1 h at 4°C at 60 rpm in a rocker. The supernatant mixture was loaded on to the column and the flow-through was collected. Finally, the column was washed with ten volumes of wash buffer (300 mM NaCl, 50 mM NaHPO_4_, 20 mM imidazole, and 15 % glycerol). The recombinant peptide OsASR2, was eluted with elution buffer according to the manufacturer’s instructions (Thermo, Rockford, USA).

**Electrophoretic Mobility Shift Assay**

The probe containing GT-1 element was synthesized artificially and labelled with dUTP using terminal deoxynucleotidyl transferase (Thermo, Rockford, USA) and incubated with the purified OsASR2 protein (1 μg per reaction). Subsequently, EMSA was conducted using a LightShift Chemiluminescent EMSA kit according to the manufacturer’s instructions (Thermo, Rockford, USA). In parallel, to determine binding specificity, competition experiments were carried out with unlabeled or mutated probe added to the reaction in 5- to 100-fold molar excess over the labeled probe. After a 20 min incubation at room temperature, the completed reactions were separated using PAGE, and transferred to nylon membrane and subjected to chemiluminescence examination.

**Chromatin Immunoprecipitation Assay**

The rice *Os2H16* promoter and *OsASR2* were co-expressed in tobacco leaves for five days. Then, 1 g of tobacco leaves was washed twice in cold PBS buffer before placing it into a plastic basket that was placed into formaldehyde at a final concentration of 1%. Vacuum was applied for 10 min, and the samples remained in this buffer for another 10 min. Subsequently, the leaf material was transferred to PBS buffer containing 0.3 M glycine. Vacuum was again applied for 5 min with subsequent incubation for 5 min. Leaves were washed twice in cold PBS buffer, dried with paper towels, frozen and stored in liquid nitrogen till further processing. Whole-cell extracts were prepared as described (Hanaoka *et al.*, 2008). ChIP was conducted using a ProFound^TM^ c-Myc Tag IP/Co-IP kit according to the manufacturer’s instructions (Thermal, Rockford, USA). The supernatant was collected, 20 μL of 5 M NaCl was added to the pooled eluate (500 μL), and the mixture was incubated at 65°C for 5 h for reversion of cross-linking. To obtain input DNA, approximately one-tenth of the volume of whole-cell extract was subjected to reversion. After ethanol precipitation, washing with cold 70% ethanol, and drying, the pellet was dissolved in water and stored at -80°C for later use.

**ChIP-qPCR**

The prepared DNA in ChIP was applied for qPCR using respective primers (see Table S1) in an UltraSYBR Mixture (CWBIO, Beijing, China) with an ABI QuantStudio^TM^ 6 Flex real-time PCR detection system. PCR reactions were performed in triplicated for each sample, and the expression levels were normalized to the input sample for enrichment detection. The fold enrichment was calculated against the region without the GT-1 *cis*-element.

**Subcellular Localization of OsASR2**

GFP was used as a reporter to investigate the subcellular localization of OsASR2 *in planta*. The full length of OsASR2 was cloned in frame with green fluorescent protein in the vector *pEarleyGate103* (Invitrogen, CA, USA), generating pEarleyGate103-OsASR2. The construct described above was introduced into *A. tumefaciens* strain GV3101 and transiently expressed in *N. benthamiana* leaves. Two days after infiltration, the treated leaves were immersed in 100 ng/mL 4ˊ, 6-diamidino-2-phenylindole (DAPI) at room temperature for 15 min and observed using Nikon ECLIPSE 90i fluorescence microscope (Nikon Microsystems, Tokyo, Japan).

**Trans-Activation Activity Analysis in Yeast**

The full-length *OsASR2* coding region was amplified by PCR with primers listed in Table S1 and cloned into *pGBKT7* (Clontech, Mountain View, CA, USA). The recombinant plasmid was transformed into the yeast strain AH109, which harbors the *HIS3* reporter gene. HIS3 activity was confirmed by growth on solid medium lacking histidine.
